# Integration of Motor Learning Principles Into Virtual Reality Interventions for Individuals With Cerebral Palsy: Systematic Review

**DOI:** 10.2196/23822

**Published:** 2021-04-07

**Authors:** Marika Demers, Karen Fung, Sandeep K Subramanian, Martin Lemay, Maxime T Robert

**Affiliations:** 1 Division of Biokinesiology and Physical Therapy University of Southern California Los Angeles, CA United States; 2 Centre for Interdisciplinary Research in Rehabilitation and Social Integration Department of Rehabilitation, Faculty of Medicine Université Laval Quebec, QC Canada; 3 Department of Physical Therapy School of Health Professions UT Health San Antonio San Antonio, TX United States; 4 Centre de Réadaptation Marie Enfant Centre de Recherche du CHU Sainte Justine Montreal, QC Canada; 5 Département des Sciences de l'Activité Physique Université du Québec à Montréal Montreal, QC Canada

**Keywords:** virtual rehabilitation, upper limb, brain damage, feedback, active video games, learning

## Abstract

**Background:**

Increasing evidence supports the use of virtual reality systems to improve upper limb motor functions in individuals with cerebral palsy. While virtual reality offers the possibility to include key components to promote motor learning, it remains unclear if and how motor learning principles are incorporated into the development of rehabilitation interventions using virtual reality.

**Objective:**

The objective of this study was to determine the extent to which motor learning principles are integrated into virtual reality interventions targeting upper limb function in individuals with cerebral palsy.

**Methods:**

A systematic review was conducted according to the PRISMA (Preferred Reporting Items for Systematic Reviews and Meta-Analyses) guidelines. The search was performed in 10 databases using a combination of keywords related to cerebral palsy, virtual reality, video games, and rehabilitation. Studies were divided into 2 categories: commercial video game platforms and devices and custom virtual reality systems. Study quality was assessed using the modified Downs and Black checklist.

**Results:**

The initial search yielded 1497 publications. A total of 26 studies from 30 publications were included, with most studies classified as “fair” according to the modified Downs and Black checklist. The majority of studies provided enhanced feedback and variable practice and used functionally relevant and motivating virtual tasks. The dosage varied greatly (total training time ranged from 300 to 3360 minutes), with only 6 studies reporting the number of movement repetitions per session. The difficulty progression and the assessment of skills retention and transfer were poorly incorporated, especially for the commercial video games.

**Conclusions:**

Motor learning principles should be better integrated into the development of future virtual reality systems for optimal upper limb motor recovery in individuals with cerebral palsy.

**Trial Registration:**

PROSPERO International Prospective Register of Systematic Reviews CRD42020151982; https://www.crd.york.ac.uk/prospero/display_record.php?ID=CRD42020151982

## Introduction

Cerebral palsy (CP) is the most common neuromotor disorder in children, with a prevalence ranging from 1.5 to 2.5 per 1000 births [1-3], that continues throughout adulthood. It is defined as “a group of permanent disorders of the development of movement and posture, causing activity limitation, that are attributed to non-progressive disturbances that occurred in the developing fetal or infant brain” [4]. Due to structural brain abnormalities, individuals with CP have a wide range of sensorimotor impairments, including muscle tone disorders [5], sensory deficits [6-8], and deficits in interjoint coordination [9], motor execution, and planning [10,11]. These impairments ultimately lead to altered upper limb function. Due to the important contribution of the arms and hands in daily activities, deficits in upper limb functions ultimately may result in a poorer quality of life [12].

To improve both motor function and quality of life in individuals with CP, moderate to strong evidence supports motor learning-based approaches in rehabilitation [13,14]. Motor learning is defined as a set of processes based on principles of neuroplasticity associated with practice or experience that lead to relatively permanent motor changes [15,16]. The brain’s inherent ability to organize itself and to form new connections between neurons can be exploited with therapeutic rehabilitation approaches based on motor learning principles [17,18]. Key components of motor learning–based approaches include but are not limited to (1) intensive rehabilitation interventions involving a high number of task repetitions, (2) progressive incremental increases in task difficulty, and (3) salient interventions to enhance motivation and engagement in therapy [15,19]. The provision of extrinsic feedback on either the movement quality or the motor performance can also promote motor learning [20]. Extrinsic feedback can compensate for reduced availability and/or processing ability of intrinsic feedback in individuals with CP. However, provision of extrinsic feedback is often not individualized to the motor abilities of each individual nor standardized [20]. Additional challenges to incorporating motor learning principles in clinical practice include (1) accountability for the heterogeneity and severity of the sensorimotor impairments observed in CP, (2) personalization of interventions based on the individual’s needs or goals, and (3) delivery of exercises that are both challenging and enjoyable [21].

Use of technology has helped to address some of the aforementioned challenges with rehabilitation interventions. Technology-based interventions including virtual reality and active video games have gained popularity in rehabilitation, with many systems designed to encourage upper limb function [22-24]. Virtual rehabilitation refers to interventions that are built on virtual reality platforms to meet rehabilitation goals. It encompasses a continuum of technologies of different types and technical complexities ranging from fully immersive 3D virtual reality viewed using commercially available head-mounted displays (eg, Oculus Rift; Facebook Technologies, LLC) to active video games or exergames (eg, commercial video games used for rehabilitation purposes or active video games primarily used for physical activity) [25]. Rehabilitation using virtual reality–based platforms offers the possibility to deliver high-intensity training in a multimodal training environment [26]. Virtual reality interventions also provide a unique opportunity to customize and standardize the levels of task difficulty by modifying the spatial and temporal constraints and the cognitive challenge. Feedback provision on the individual abilities and delivery modes can be easily manipulated. The task outcome and quality can be automatically recorded, which is useful to both clinicians and researchers [27]. Virtual reality has been shown to be safe and ecologically valid for the rehabilitation of individuals with CP [28-30]. The novelty of virtual reality technology and the interactive and engaging gaming characteristics are key components that provide a joyful training environment to sustain and enhance motivation to treatment [31,32]. Therefore, the attributes of virtual environments such as motivation, repetitive practice, and enhanced feedback make them an ideal modality to facilitate the incorporation of motor learning principles into the treatment of individuals with CP.

Several systematic reviews and one meta-analysis investigated the impact of virtual reality interventions on upper limb motor recovery in children and adolescents with CP [13,22,27,33-35]. Their results suggest, to an extent, that virtual reality can be effective and motivating for children with CP. Another literature review specifically looked at the effectiveness of virtual reality on motor learning in children with CP [36]. The results support virtual reality interventions to improve motor learning and encourage skill transfer to real-life situations. A current knowledge gap in the literature relates to which exact motor learning principles are incorporated into virtual reality–based platforms used for upper limb rehabilitation in individuals with CP (children, adolescents, and adults). The limited incorporation of key motor learning principles in therapy (eg, treatment intensity and specificity, feedback provision and delivery, and difficulty progression) could explain differences observed between studies and also limit the potential for motor learning in individuals with CP. This systematic review aims to identify the extent to which motor learning principles are integrated into virtual reality interventions targeting upper limb function in individuals with CP. The incorporation of motor learning principles will be identified for commercial video game platforms and devices and custom virtual reality systems for rehabilitation to help guide clinical decision making.

## Methods

### Protocol and Registration

This systematic review followed the PRISMA (Preferred Reporting Items for Systematic Reviews and Meta-Analyses) guidelines, and results are reported using the PRISMA checklist [37]. The protocol for this systematic review was registered on PROSPERO (CRD42020151982).

### Eligibility Criteria

We included publications related to the effectiveness of virtual reality interventions on upper limb sensorimotor function at the levels of body function/structure or activity limitations according to the International Classification of Functioning, Disability and Health in individuals with CP. We only included peer-reviewed publications in English or French in which the majority of the sample included individuals with CP (children or adults). We excluded publications that (1) used solely qualitative methodologies (eg, focus groups and interviews), (2) focused on the development of the virtual reality intervention, (3) used virtual reality as an assessment tool, or (4) were reviews, meta-analyses, or commentaries.

### Information Sources

The following electronic databases were searched on August 22, 2019, and updated on July 5, 2020, using a combination of keywords related to CP, rehabilitation, video games, and virtual reality: MEDLINE, Embase, CINAHL, Web of Science, Google Scholar, OTseeker, Physiotherapy Evidence Database, IEEE Xplore, Scopus, and Cochrane Library, and the Cochrane Central Register of Controlled Trials. The reference lists of articles of interest were also searched for additional references. The search strategy was developed for the MEDLINE database and adapted for other databases. Various combinations of keywords and Medical Subject Headings (MeSH) or Embase subject headings (Emtree) related to virtual reality or video games, rehabilitation, and CP were used (see Multimedia Appendix 1 for the detailed search strategy). No time limit regarding the date of publication was applied to the search strategy.

### Search and Study Selection

The search strategy was executed by one researcher (MD), and all publications from each database were extracted using citation management software (Endnote X9; Clarivate). Any duplications were removed. Titles and abstracts were screened independently by two researchers (MD and KF) based on the inclusion and exclusion criteria. For all potential eligible studies, full texts were retrieved, and eligibility was assessed by the same two researchers. Any conflict was resolved by discussion.

### Data Collection Process and Data Items

For all publications meeting the inclusion criteria, one researcher (MD) extracted the following information into an Excel (Microsoft Inc) spreadsheet: author/date, study design, participants, sample size, virtual reality system, delivery method, number of movement repetitions, intensity, task specificity, difficulty progression, type of practice, type of feedback, feedback delivery schedule, motivation, motor recovery outcome measures, changes in upper limb motor function (body function/structure or activity limitation levels), assessment of skills retention, and assessment of transfer of skills. Another researcher (KF) validated the data extraction by reading all included publications and confirming that the data extracted were accurate and complete. Publications presenting the results from the same group of participants were considered a single study and the results were extracted together. Since there is a lack of clear definition of motor learning principles in CP, we used the definition and key descriptors of Maier et al [38] for dosage, type of practice, feedback provision, and task specificity. For treatment intensity and duration, the number of movement repetitions was extracted and the number of minutes of treatment was computed using the treatment duration, frequency, and number of weeks of the intervention. Data were analyzed separately for commercial video game platforms and devices and custom virtual reality systems for rehabilitation. Commercial video game platforms and devices included salon game consoles and commercially available applications on a tablet. Custom virtual reality systems for rehabilitation included commercially available or custom virtual reality software programmed for rehabilitation purposes. Studies using special hardware (eg, instrumented gloves or robotic devices) or repurposed commercial gaming hardware (eg, the Microsoft Kinect camera) were included in the custom virtual reality software category.

### Risk of Bias in Individual Studies

Two authors (SKS and MTR) analyzed the quality of each study, and conflicts were resolved by discussion. The 27-item modified version [39] of the original Downs and Black checklist [40] helped assess the quality of the included randomized and nonrandomized studies. The overall quality of the research was scored out of 28 based on the following criteria: reporting, internal validity, power, and external validity. Scores on the modified Downs and Black checklist were classified as “excellent” (scores of 24-28), “good” (scores of 19-23), “fair” (scores of 14-18), or “poor” (scores ≤13) [41]. The Downs and Black checklist was chosen over other measures, such as the Effective Public Health Practice Project [42], because it considers sample size in the total score calculation and enables studies to be quantitatively classified into different categories based on the total score. The Downs and Black checklist has previously been used in studies involving virtual reality interventions for upper limb rehabilitation [43].

## Results

**Figure 1:**
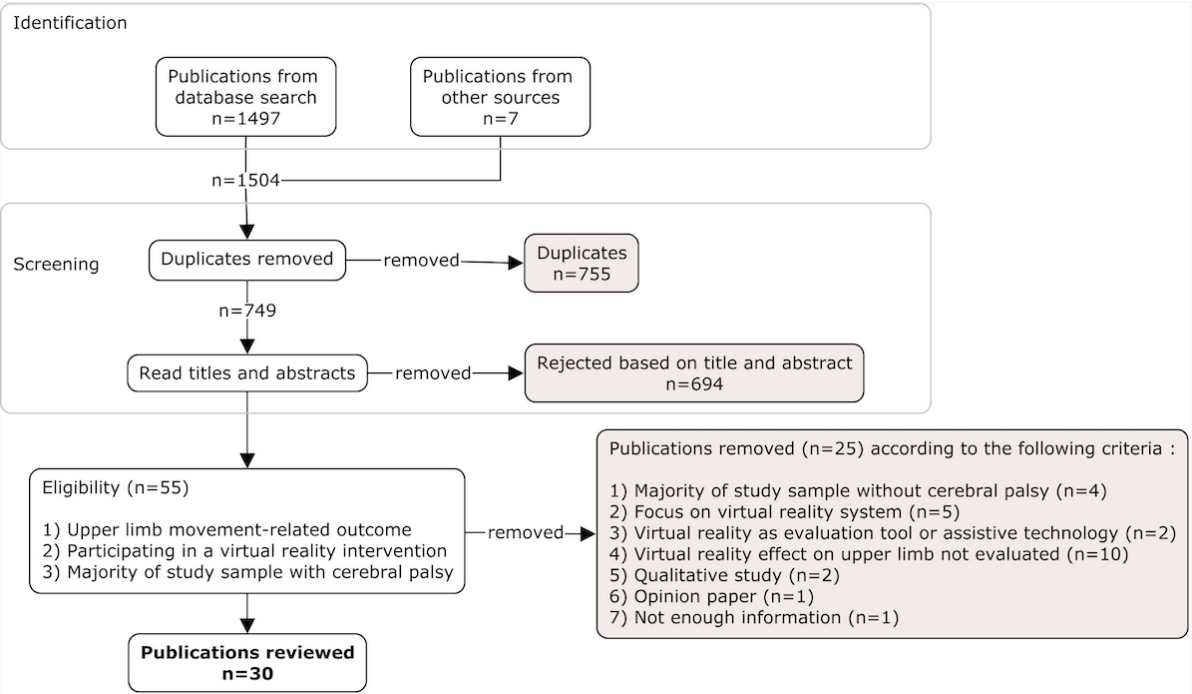
PRISMA (Preferred Reporting Items for Systematic Reviews and Meta-Analyses) flow chart of study selection.

The database search yielded 1497 publications, and 749 publications were screened for eligibility after duplicates were removed. After full-text review, 26 studies from 30 publications were included (see Figure 1 for flow diagram and reasons for exclusion).

### Quality Assessment

The quality ratings of the publications based on the Downs and Black checklist are shown in Table 1. Only 17 studies were rated because the remaining studies were either case studies or short papers. Of the 17 studies, 2 were rated as good, 14 as fair, and 1 as poor.

**Table 1:** Quality assessment of the reviewed studies based on the 27-item modified version [39] of the Downs and Black checklist [40].

First author and publication year	Downs and Black score/28	Quality of study
Avcil et al, 2020 [44]	21	Good
Bedair et al, 2016 [45]	16	Fair
Chen et al, 2007 [46]	15	Fair
Sharan et al, 2012 [47]	13	Fair
El-Shamy, 2018 [48]	16	Fair
Fluet et al, 2009 [49]	16	Fair
Fluet et al, 2010 [50]	15	Fair
Hernández et al, 2018 [51]	17	Fair
Jannink et al, 2008 [52]	14	Fair
Kassee et al, 2017 [53]	17	Fair
Odle et al, 2009 [54]	14	Fair
Rostami et al, 2012 [55]	18	Fair
Sahin et al, 2020 [56]	19	Good
Sandlund et al, 2014 [57]	13	Fair
Turconi et al, 2016 [58]	18	Fair
Weightman et al, 2011 [59]	12	Poor
Winkels et al, 2013 [60]	13	Fair

### Characteristics of the Virtual Reality Systems

Of the 26 studies, 9 (35%) used commercial video game platforms and devices, such as the Nintendo Wii, the Sony PlayStation 2 or 3, Xbox consoles (Microsoft Corporation), or commercial applications [44,45,47,52,53,58,60-62]. Custom virtual reality systems for rehabilitation were used in 17 studies (65% [46,48,50,51,54-56,59,63-71]). Commercially available virtual reality systems designed for rehabilitation purposes were used in 5 studies (19%): ArmeoSpring (Hocoma) combined with virtual games [48]; E-Link Evaluation and Exercise System (Biometrics Ltd) [55]; IREX (GestureTek Health) [66]; Timocco [64]; and YouGrabber (YouRehab) [67]. Seven studies (27%) used custom-based games combined with commercially available accessories (ie, Microsoft Kinect camera [56,68,72], webcam [54], force-feedback motion controller [51], or instrumented gloves [69,70]). Custom virtual reality systems designed for rehabilitation research purposes were used in 4 studies (15%) [49,50,59,65,71]. Display media used to view the virtual environments included stereoscopic glasses to enable 3D media display view [49,50,71] and television or computer monitors to view 2D virtual environments with various 3D rendering features (ie, shadow, drop lines, etc). Virtual environments ranged from a simple display of reaching targets in a 2D plane to a detailed replication of real-life environments, such as a tennis court or a kitchen. None of the studies used immersive virtual reality through a head-mounted display.

### Study Characteristics

**Figure 2:**
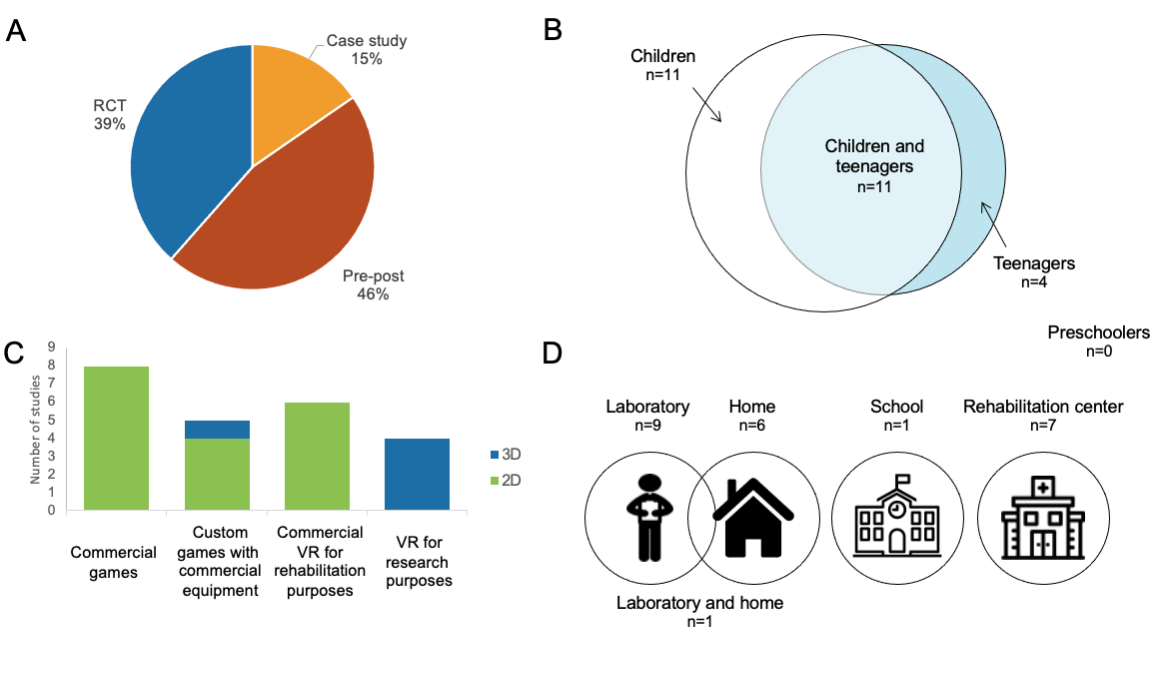
Characteristics of the reviewed studies according to (A) study design, (B) participants’ age groups (preschoolers: aged 0-4 years; children: aged 4-12 years; teenagers: aged 13-18 years), (C) virtual reality (VR) system type, and (D) delivery environment. RCT: randomized controlled trial.

The study settings and targeted participants varied greatly. Sample sizes ranged from 1 to 30 participants in the intervention group, with 12 studies (46%) having a sample size of 5 participants or fewer. While our search strategy did not exclude studies conducted in adults with CP, none of the studies targeted participants over 18 years of age (see Figure 2 for detailed study characteristics). All studies targeted school-aged children (over 4 years of age) or adolescents. In 17 studies (65%), treatment was delivered in a laboratory setting [46,48,50,55,58,61,65-67,71] or a rehabilitation center [45,47,51,52,54,60,68]. In 6 studies (23%), the virtual reality–based intervention was delivered at home using either telerehabilitation technologies (ie, videoconferencing and remote monitoring [64,69,70]) or a prescribed exercise program [53,57,59].

### Motor Learning Principles in Studies Using Commercial Video Game Platforms and Devices

**Figure 3:**
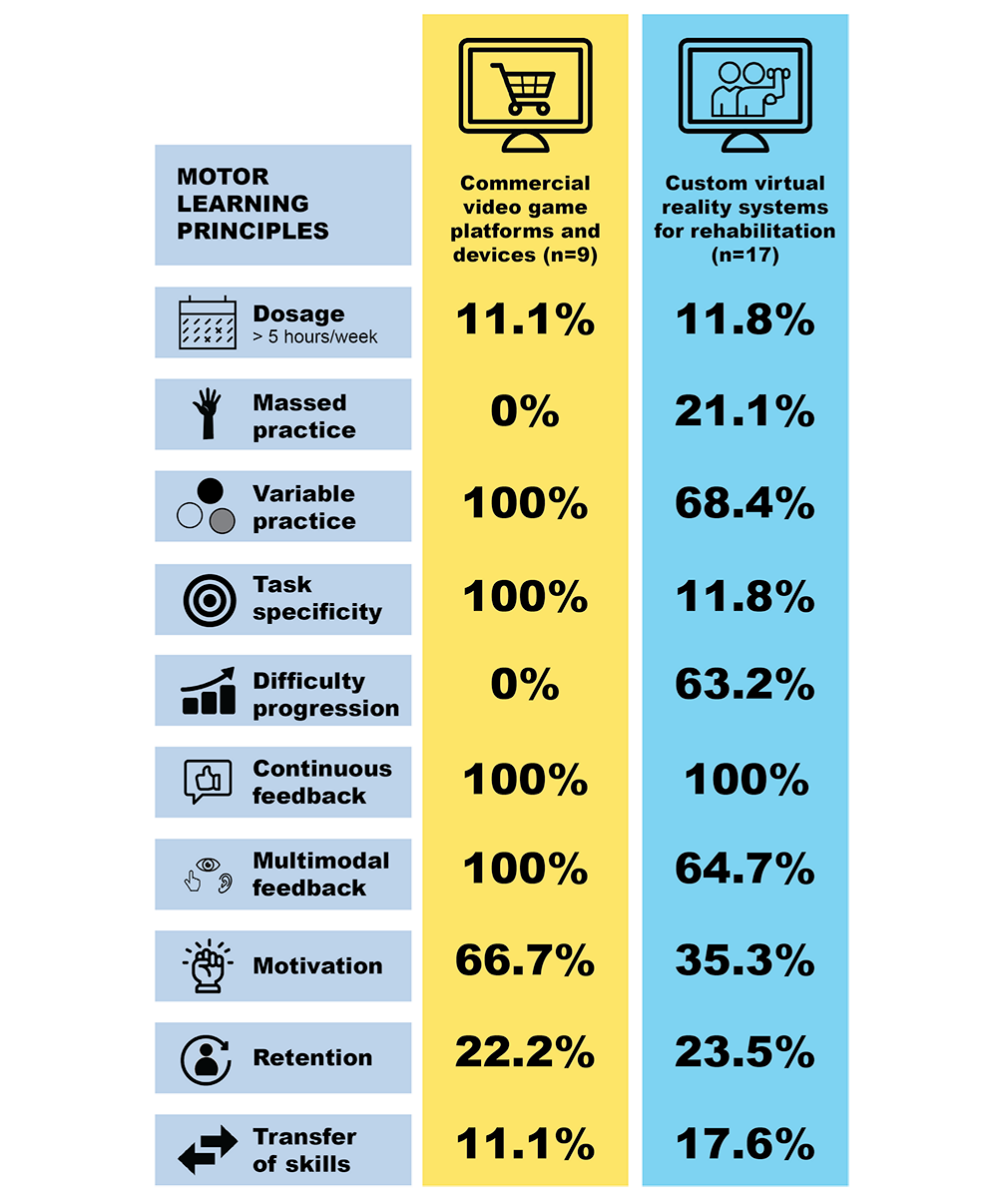
Percentage of commercial video game platforms and devices and custom virtual reality systems for rehabilitation integrating the principles of motor learning.

Among the 9 studies (35%) using commercial video game platforms and devices, the length of sessions varied from 20 to 60 minutes (Figure 3 and Multimedia Appendix 1). Frequency ranged from 2 to 5 sessions per week for 3 to 12 weeks, with a total treatment time ranging from 360 to 1440 minutes. Only Kassee et al [53] discussed the number of movement repetitions, suggesting that the number of movement repetitions in the virtual reality group was comparable with that in the resistance training group (144 repetitions per session). Jannink et al [52] indicated that the intensity level was moderate but did not define how intensity was measured in their study. Task-specific training, in which movements were goal-oriented or relevant for activities of daily living, was used in all 9 studies and the majority simulated competitive sports. Examples of task-specific training included playing various sports (eg, tennis, bowling, sword fighting), controlling a moving car, piloting a spaceship, and catching falling balls. Variable practice was delivered in all studies using commercial video game platforms and devices by using frequent changes of tasks. However, none of the studies clearly specified the type of practice offered with the intervention. None of the publications reported whether the games were adapted to match the participant’s capacity and, if so, how they were adapted and how the difficulty levels were changed.

Visual and auditory feedback were provided in all studies as a display of total score and/or reward sounds. Additional haptic feedback (vibration) was offered by a motion controller held in the most-affected hand in 2 studies [44,53]. All studies delivered continuous feedback. For virtual interventions, an example of continuous feedback is the provision of knowledge of results after each trial, such as the rate of success or total score. Motivation with the intervention was assessed in 6 studies (23%) using questionnaires (eg, a visual analog scale for motivation and a participant and parent feedback questionnaire), therapists’ observations, or semistructured interviews. High motivation to practice in a virtual environment was reported [47,52,53,58,60,62]. However, Sandlund et al [57] noted that children’s interest in gaming faded somewhat over time. Winkels et al [60] observed that motivation varied between games and children. In 2 studies, greater motivation was observed in the Wii training group than in a control group [47,53]. Retention of motor skills was assessed in only 2 studies [53,61]; the results of both studies suggested that motor skills were retained 4 weeks after the intervention. Transfer of skills was only assessed in 1 study, with the skills shown to be transferred to a similar reaching task [57].

### Motor Learning Principles in Studies Using Custom Virtual Reality Systems for Rehabilitation

In all 17 studies (65%) using custom virtual reality systems for rehabilitation, the treatment frequency and intervention dosage were clearly reported, with the total training time ranging from 300 to 3360 minutes. The number of movement repetitions was reported in only 5 studies (range of 45 to 550 repetitions per session) [46,50,51,59,72]. Nine studies (35%) delivered task-specific training with functionally relevant tasks [46,48-50,56,64,66-68,71]. Thirteen studies (50%) delivered variable practice, with 4 studies (15%) also delivering massed practice (eg, minimal time between sessions and large number of movement repetitions to a single target) [46,49-51,59] and 1 study (4%) using random practice (ie, randomized tasks to maintain patients' interest) [70]. Two studies (8%) delivered constant practice [64,65], while the remaining studies did not specify the type of practice used. Task difficulty was increasingly progressed in 12 studies [46,48,50,51,55,59,65,67-71] by different methods, including a modification of the ranges of motion required to accomplish the task, an increase or decrease in the amount of assistive and/or resistive force, and a change in the speed, accuracy, or target characteristics. Difficulty levels were progressed by the system using an algorithm, based on the judgment of a therapist according to task success, according to difficulties reported by the participants, or based on preset difficulty levels (eg, easy, medium, hard).

Multisensory feedback that combined visual and auditory and/or haptic feedback was offered in 11 studies (42%) [46,48-51,55,59,63,64,70,71,73]. Six studies (23%) provided only visual feedback [54,56,64-66,68]. Feedback was delivered continuously in all studies, but 1 study did not report on feedback frequency [51]. Motivation was assessed in 6 studies (23%) using subjective assessment or semistructured interviews [46,50,51,64,69,71]. The results suggested that the virtual environments were motivating, but Chen et al [46] and Hernández et al [51] reported that motivation levels were highly variable from one child to another, ranging from low to high. Retention of motor skills was assessed in only 4 studies [46,55,65,66]; in 3 of the 4 studies, the motor skills gained by the virtual reality intervention were maintained or improved at 1 to 3 months [46,55,66]. Three studies assessed the transfer of skills [51,59,66]. Hernández et al [51] reported that all children made significant progress on their self-selected goals, which largely targeted activities of daily living and leisure. In another study [66], 66% of the children showed a transfer of skills to a similar reach-to-grasp task for all kinematic variables. Weightman et al [59] noticed improvements in activities of daily living not directly targeted by the intervention.

## Discussion

### Principal Findings

The objective of this systematic review was to examine the extent to which motor learning principles are integrated into virtual reality interventions in individuals with CP. A total of 26 studies met the inclusion criteria, of which 9 used commercial video game platforms and devices and 17 developed custom virtual reality systems for rehabilitation. Overall, the studies were considered fair based on the Downs and Black checklist, given that the majority of them were small pilot or proof-of-concept studies. Nonetheless, the novelty of this review is that virtual reality is well-suited to incorporate motor learning principles into rehabilitation interventions targeted at children and adolescents with CP. Proper integration of motor learning principles is important, as demonstrated by the fact that the most effective therapies (ie, constraint-induced movement therapy and bimanual therapy) for improving upper limb motor functions are themselves based on motor learning principles and principles of neuroplasticity [13,74,75]. Enhanced feedback provision, variable practice, task specificity, and motivation were the motor learning principles most frequently adopted in virtual reality interventions for children and adolescents with CP. Dosage varied greatly from one study to another with only a few studies reporting the number of movement repetitions per session. The application of some principles (ie, difficulty progression and assessment of skills retention and transfer) were poorly integrated, especially in commercial video game platforms and devices.

It is widely accepted that rehabilitation interventions should be delivered at a high intensity (dose, frequency, and duration of training) to engage neuroplastic mechanisms [15]. Unfortunately, our results showed that many of the reviewed studies did not provide sufficient information on the number of repetitions performed per session. Although intensive and repetitive practice is important, repetitive motor activity alone is insufficient to induce experience-dependent plasticity [76]. Virtual reality enables the possibility to deliver high-intensity practice of engaging and meaningful tasks along with relevant feedback. This promotes a problem-solving approach known to be useful for enhanced rehabilitation outcomes. Additionally, a close collaboration between game developers, academia, and clinicians in the development of both commercial and custom virtual reality systems would help identify the clinical needs and optimize virtual reality interventions for individuals with CP [77].

### Extrinsic Feedback

In both the commercial and custom virtual reality systems, feedback provision was well integrated. Feedback plays a crucial role to enhance motor learning and motivation level [78,79]. All studies reported providing feedback at a continuous frequency, yet provision of continuous feedback is often not optimal for motor learning. Continuous feedback limits the opportunity for learning to occur through exploration and increases the dependence of the user on the feedback to improve specific goals, thereby affecting the ability of learning to detect and correct errors [80]. While continuous feedback may increase skills acquisition, retention of improvements is rarely sustained over time beyond the cue that prompted them (ie, feedback). Alternatively, provision of faded feedback (ie, gradual decrease in feedback provision as the learner improves) or self-controlled feedback may encourage the participant to explore and internalize new movement patterns, thus increasing the retention of these newly acquired skills [81,82]. Based on the limited evidence on the feedback modality that should be prioritized, a combination of multimodal feedback (ie, visual, auditory, and haptic) is proposed to be more effective for improving motor performance [80,83]. All studies using commercial video game platforms and devices implemented multimodal feedback. In contrast, approximately one-third of the studies using custom virtual reality systems delivered solely visual feedback. This could be because of the technical complexity required to program multimodal feedback and the lack of knowledge about which feedback modality should be provided in rehabilitation settings. Feedback should be implemented in a structured manner considering the individual capacities and errors made and thus allow the progression of difficulty throughout the intervention period. The use of theoretical frameworks, such as the framework developed by Schüler et al [84], can help designers and researchers to identify the beneficial components of virtual reality systems for specific treatment goals.

### Progression of Difficulty

Constant progression of difficulty promotes motor learning because an individual’s abilities are considered within the conditions of a specific learning experience. According to the Challenge Point Framework [85], learning occurs through active problem solving. Errors committed during motor learning are necessary to both improve movement behavior and provoke neuroplasticity [86]. The majority of custom virtual reality systems for rehabilitation successfully implemented this concept. Unfortunately, all 9 studies using commercial video game platforms and devices did not report on the progression of difficulty, which does not enable assessment of whether the participants in these studies were appropriately challenged. This is somewhat surprising, since game developers generally integrate difficulty progression to maintain players’ enjoyment. Commercial video game platforms and devices can have limited therapeutic value for individuals with physical disabilities because they are designed for nondisabled populations [87]. Depending on the severity of the sensorimotor impairments, individuals with CP may not achieve the minimum threshold to progress through the difficulty levels in a given game. The concept of progression of difficulty is nonnegligible, as it may have a negative impact if a task is deemed too difficult. Thus, the strong association between challenge and motivation highlights the importance of delivering interventions at an appropriate difficulty level.

### Motivation

Motivation is a critical element of rehabilitation, especially in the pediatric population [88,89]. A lack of motivation both increases activity limitations and decreases the child’s participation, thus hindering adherence to treatment [88]. Higher levels of motivation help lead an individual toward satisfying their specific needs and achieving goals in a persistent manner [90]. Given that most commercial games have high production value and include gamification elements to promote motivation and volition [91], most of the studies using commercial video game platforms and devices reported a high level of motivation. However, it is also possible that while commercial video games may be perceived as motivating and fun, they might still be too difficult for participants with CP. Driving elements of motivation include appropriately challenging tasks, game variability, setting realistic goals, and aspects of competition such as a reward system [92]. Unfortunately, these elements were often not incorporated into custom virtual reality systems for rehabilitation. Reasons contributing to these findings could include a lack of financial resources available in the development of a game and/or the lack of collaboration between game developers, clinicians, and end users. Future studies must comprehensively assess and report motivation levels and whether or not they were sustained in the long term. Moreover, strategies used to drive motivation should also be reported to completely understand the utility of such strategies.

### Retention and Transfer of Skills

Two of the most important principles of motor learning pertain to how much the improvements are retained over an extended period and how much of the newly acquired skill can be transferred to performance of a similar task [93,94]. The majority of the studies reviewed, regardless of the type of virtual reality used, did not examine these principles in depth. A few studies, however, referenced the potential to retain the newly acquired upper limb skills [46,55,61,66], as well as the possibility to transfer motor skills to real-life activities [51,57,66,73]. Virtual reality is well suited to address important factors that potentially encourage retention and transfer of skills, such as high enjoyment level, physical fidelity of the practiced movement, and high repetitions. Thus, retention is an important factor that must be addressed in future studies.

### Type of Practice

Relatively few details were provided regarding the practice conditions used for studies with the commercial video game platforms and devices. For the custom virtual reality systems, 4 studies reported using massed practice. The beneficial impact of massed or distributed practice on learning is not clear and is likely related to contextual factors such as the nature (discreet vs continuous) and the difficulty of the task or the expertise of the participants. A pilot study conducted in healthy participants that compared massed practice with distributed practice in a virtual reality setting did not report any significant difference between these 2 types of practice [95]. Thirteen studies reported using variable practice, while only 1 study reported using random practice. Both variable and random practice tend to negatively affect short-term performance but often have a positive long-term impact on skill retention and transfer [96]. Variable and random practice are notably more cognitively engaging [97], more challenging, and improve generalization and adaptability, as observed when performing a novel variation of a task [85]. Our results show that many interventions do not take full advantage of increasing variability within trials to optimize skill retention and transfer. The concept of variability was first introduced by Bernstein [98], who emphasized that the success of practice relies on the process of solving a problem repetitively. To this day, this concept remains relevant and should be considered when developing virtual reality interventions. Nonetheless, it should be noted that in some contexts, blocked and constant practice might be more suitable, notably in younger children or in difficult tasks [99-101]. Therefore, virtual reality software should be flexible enough to allow the clinician to adjust the variability within a block of trials in order to maximize motor learning.

### Limitations

Overall, our interpretation of the results of these studies was limited by the available information provided in the publications. For example, inconsistent reporting of the type of feedback and delivery schedule hindered our ability to conclude whether feedback was provided in the form of knowledge of performance or knowledge of results [102]. In other words, our review was constrained by the level of detail in the studies’ methodology sections, which often mirrored the quality of the studies. Thus, no conclusion was drawn on the effectiveness of virtual reality in rehabilitation, as it was beyond the scope of this review.

### Conclusions

This review demonstrates the current integration of select principles of motor learning into commercial video game platforms and devices and custom virtual reality systems designed for upper limb motor recovery. Overall, motor learning principles are not yet being fully integrated into virtual reality systems, especially into commercial video game platforms and devices, because the target audience is not individuals with disabilities. Custom virtual reality systems are better tailored to the needs of individuals with CP and provide an experience better adapted to the capacity of individuals in term of difficulty. However, the custom virtual reality systems used in this review were not as engaging as commercial video game platforms and devices nor did they provide multimodal feedback. Nonetheless, designing an intervention using multimodal feedback may be feasible with the proper resources. The integration of motor learning principles into such a system would help maximize its efficiency and offer a cost-effective intervention to supplement standard treatments in the clinical setting. Future research should provide detailed methodology on the extent to which motor learning principles are integrated to help evaluate the efficacy of video game platforms and devices and virtual reality systems in improving upper limb function.

## Appendix

Multimedia Appendix 1 Detailed search strategy.

## Abbreviations

CP: cerebral palsy
MeSH: Medical Subject Headings
PRISMA: Preferred Reporting Items for Systematic Reviews and Meta-Analyses

## References

[1] Bishop DVM (2010). Which neurodevelopmental disorders get researched and why?. PLoS One.

[2] Paneth N, Hong T, Korzeniewski S (2006). The descriptive epidemiology of cerebral palsy. Clin Perinatol.

[3] Oskoui M, Coutinho F, Dykeman J, Jetté Nathalie, Pringsheim T (2013). An update on the prevalence of cerebral palsy: a systematic review and meta-analysis. Dev Med Child Neurol.

[4] Rosenbaum Peter, Paneth Nigel, Leviton Alan, Goldstein Murray, Bax Martin, Damiano Diane, Dan Bernard, Jacobsson Bo (2007). A report: the definition and classification of cerebral palsy April 2006. Dev Med Child Neurol Suppl.

[5] Shortland A (2009). Muscle deficits in cerebral palsy and early loss of mobility: can we learn something from our elders?. Dev Med Child Neurol.

[6] Robert MT, Guberek R, Sveistrup H, Levin MF (2013). Motor learning in children with hemiplegic cerebral palsy and the role of sensation in short-term motor training of goal-directed reaching. Dev Med Child Neurol.

[7] Auld ML, Boyd RN, Moseley GL, Ware RS, Johnston LM (2012). Impact of Tactile Dysfunction on Upper-Limb Motor Performance in Children With Unilateral Cerebral Palsy. Arch Phys Med Rehabil.

[8] Gupta D, Barachant A, Gordon AM, Ferre C, Kuo H, Carmel JB, Friel KM (2017). Effect of sensory and motor connectivity on hand function in pediatric hemiplegia. Ann Neurol.

[9] Eliasson A, Gordon AM (2000). Impaired force coordination during object release in children with hemiplegic cerebral palsy. Dev Med Child Neurol.

[10] Kurz MJ, Becker KM, Heinrichs-Graham E, Wilson TW (2014). Neurophysiological abnormalities in the sensorimotor cortices during the motor planning and movement execution stages of children with cerebral palsy. Dev Med Child Neurol.

[11] Steenbergen B, Gordon AM (2006). Activity limitation in hemiplegic cerebral palsy: evidence for disorders in motor planning. Dev Med Child Neurol.

[12] Makris T, Dorstyn D, Crettenden A (2021). Quality of life in children and adolescents with cerebral palsy: a systematic review with meta-analysis. Disabil Rehabil.

[13] Novak I, Morgan C, Fahey M, Finch-Edmondson M, Galea C, Hines A, Langdon K, Namara MM, Paton MC, Popat H, Shore B, Khamis A, Stanton E, Finemore OP, Tricks A, Te Velde Anna, Dark L, Morton N, Badawi N (2020). State of the Evidence Traffic Lights 2019: Systematic Review of Interventions for Preventing and Treating Children with Cerebral Palsy. Curr Neurol Neurosci Rep.

[14] Sakzewski L, Ziviani J, Boyd RN (2014). Efficacy of upper limb therapies for unilateral cerebral palsy: a meta-analysis. Pediatrics.

[15] Kleim JA, Jones TA (2008). Principles of experience-dependent neural plasticity: implications for rehabilitation after brain damage. J Speech Lang Hear Res.

[16] Schmidt R, Lee T, Winstein C, Wulf G, Zelaznik (2018). Motor Control and Learning: A Behavioural Emphasis Sixth Edition.

[17] Winstein C, Kay D (2015). Translating the science into practice: shaping rehabilitation practice to enhance recovery after brain damage. Prog Brain Res.

[18] Krakauer J, Dietz V, Ward N. S. (2015). The applicability of motor learning to neurorehabilitation. Oxford Textbook of Neurorehabilitation. 2nd edition.

[19] Muratori LM, Lamberg EM, Quinn L, Duff SV (2013). Applying principles of motor learning and control to upper extremity rehabilitation. J Hand Ther.

[20] Robert MT, Sambasivan K, Levin MF (2017). Extrinsic feedback and upper limb motor skill learning in typically-developing children and children with cerebral palsy: Review. Restor Neurol Neurosci.

[21] Onla-or S, Winstein CJ (2008). Determining the optimal challenge point for motor skill learning in adults with moderately severe Parkinson's disease. Neurorehabil Neural Repair.

[22] Johansen T, Strøm Vegard, Simic J, Rike P (2019). Effectiveness of training with motion-controlled commercial video games on hand and arm function in young people with cerebral palsy: A systematic review and meta-analysis. J Rehabil Med.

[23] Leal AF, da Silva TD, Lopes PB, Bahadori S, de Araújo Luciano Vieira, da Costa MVB, de Moraes ?AP, Marques RH, Crocetta TB, de Abreu LC, Monteiro CBDM (2020). The use of a task through virtual reality in cerebral palsy using two different interaction devices (concrete and abstract) - a cross-sectional randomized study. J Neuroeng Rehabil.

[24] Metin Ökmen Burcu, Doğan Aslan Meryem, Nakipoğlu Yüzer Güldal Funda, Özgirgin Neşe (2019). Effect of virtual reality therapy on functional development in children with cerebral palsy: A single-blind, prospective, randomized-controlled study. Turk J Phys Med Rehabil.

[25] Weiss PL, Tirosh E, Fehlings D (2014). Role of virtual reality for cerebral palsy management. J Child Neurol.

[26] Burke JW, McNeill MDJ, Charles DK, Morrow PJ, Crosbie JH, McDonough SM (2009). Optimising engagement for stroke rehabilitation using serious games. Vis Comput.

[27] Snider L, Majnemer A, Darsaklis V (2010). Virtual reality as a therapeutic modality for children with cerebral palsy. Dev Neurorehabil.

[28] Gordon AM, Okita SY (2010). Augmenting pediatric constraint-induced movement therapy and bimanual training with video gaming technology. Technol Disabil.

[29] Rizzo A, Cohen I, Weiss P, Kim J G, Yeh S C, Zali B, Hwang J (2004). Design and development of virtual reality based perceptual-motor rehabilitation scenarios. Conf Proc IEEE Eng Med Biol Soc.

[30] Weiss P, Klinger E (2009). Moving beyond single user, local virtual environments for rehabilitation. Stud Health Technol Inform.

[31] Bryanton C, Bossé J, Brien M, McLean J, McCormick A, Sveistrup H (2006). Feasibility, motivation, and selective motor control: virtual reality compared to conventional home exercise in children with cerebral palsy. Cyberpsychol Behav.

[32] Levac D, Sveistrup H, Weiss PL, Keshner EA, Levin MF (2014). Motor Learning in Virtual Reality. Virtual Reality for Physical and Motor Rehabilitation.

[33] Rathinam C, Mohan V, Peirson J, Skinner J, Nethaji KS, Kuhn I (2019). Effectiveness of virtual reality in the treatment of hand function in children with cerebral palsy: A systematic review. J Hand Ther.

[34] Chen Y, Lee S, Howard AM (2014). Effect of virtual reality on upper extremity function in children with cerebral palsy: a meta-analysis. Pediatr Phys Ther.

[35] Galvin J, McDonald R, Catroppa C, Anderson V (2011). Does intervention using virtual reality improve upper limb function in children with neurological impairment: a systematic review of the evidence. Brain Inj.

[36] Massetti T, Silva TD, Ribeiro D (2014). Motor learning through virtual reality in cerebral palsy - a literature review. Med Express.

[37] Moher D, Liberati A, Tetzlaff J, Altman DG, PRISMA Group (2009). Preferred reporting items for systematic reviews and meta-analyses: the PRISMA statement. PLoS Med.

[38] Maier M, Rubio Ballester B, Duff A, Duarte Oller E, Verschure PFMJ (2019). Effect of Specific Over Nonspecific VR-Based Rehabilitation on Poststroke Motor Recovery: A Systematic Meta-analysis. Neurorehabil Neural Repair.

[39] Morton S, Barton CJ, Rice S, Morrissey D (2014). Risk factors and successful interventions for cricket-related low back pain: a systematic review. Br J Sports Med.

[40] Downs SH, Black N (1998). The feasibility of creating a checklist for the assessment of the methodological quality both of randomised and non-randomised studies of health care interventions. J Epidemiol Community Health.

[41] O'Connor Seán R, Tully MA, Ryan B, Bradley JM, Baxter GD, McDonough SM (2015). Failure of a numerical quality assessment scale to identify potential risk of bias in a systematic review: a comparison study. BMC Res Notes.

[42] Effective Public Health Practice Project (1998). Quality assessment tool for quantitative studies. National Collaborating Centre for Methods and Tools, McMaster University, Hamilton, Ontario.

[43] Subramanian S, Caramba S, Hernandez O, Morgan Q, Cross M, Hirschhauser C (2019). Is the Downs and Black scale a better tool to appraise the quality of the studies using virtual rehabilitation for post-stroke upper limb rehabilitation? InInternational Conference on Virtual Rehabilitation (ICVR).

[44] Avcil E, Tarakci D, Arman N, Tarakci E (2020). Upper extremity rehabilitation using video games in cerebral palsy: a randomized clinical trial. Acta Neurol Belg.

[45] Bedair R, Al-Talawy H, Shoukry K, Abdul-Raouf E (2016). Impact of virtual reality games as an adjunct treatment tool on upper extremity function of spastic hemiplegic children. Int J PharmTech Res.

[46] Chen Y, Kang L, Chuang T, Doong J, Lee S, Tsai Mei-Wun, Jeng Suh-Fang, Sung Wen-Hsu (2007). Use of virtual reality to improve upper-extremity control in children with cerebral palsy: a single-subject design. Phys Ther.

[47] Sharan D, Ajeesh PS, Rameshkumar R, Mathankumar M, Paulina RJ, Manjula M (2012). Virtual reality based therapy for post operative rehabilitation of children with cerebral palsy. Work.

[48] El-Shamy SM (2018). Efficacy of Armeo® Robotic Therapy Versus Conventional Therapy on Upper Limb Function in Children With Hemiplegic Cerebral Palsy. Am J Phys Med Rehabil.

[49] Fluet G, Qiu Q, Saleh S (2009). Robot-assisted virtual rehabilitation (NJIT-RAVR) system for children with upper extremity hemiplegia.

[50] Fluet GG, Qiu Q, Kelly D, Parikh HD, Ramirez D, Saleh S, Adamovich SV (2010). Interfacing a haptic robotic system with complex virtual environments to treat impaired upper extremity motor function in children with cerebral palsy. Dev Neurorehabil.

[51] Hernández Hamilton A, Khan A, Fay L, Roy J, Biddiss E (2018). Force Resistance Training in Hand Grasp and Arm Therapy: Feasibility of a Low-Cost Videogame Controller. Games Health J.

[52] Jannink MJA, van der Wilden Gelske J, Navis DW, Visser G, Gussinklo J, Ijzerman M (2008). A low-cost video game applied for training of upper extremity function in children with cerebral palsy: a pilot study. Cyberpsychol Behav.

[53] Kassee C, Hunt C, Holmes MW, Lloyd M (2017). Home-based Nintendo Wii training to improve upper-limb function in children ages 7 to 12 with spastic hemiplegic cerebral palsy. J Pediatr Rehabil Med.

[54] Odle B, Irving A, Foulds R (2009). Usability of an adaptable video game platform for children with cerebral palsy.

[55] Rostami HR, Arastoo AA, Nejad SJ, Mahany MK, Malamiri RA, Goharpey S (2012). Effects of modified constraint-induced movement therapy in virtual environment on upper-limb function in children with spastic hemiparetic cerebral palsy: a randomised controlled trial. NeuroRehabilitation.

[56] Şahin S, Köse Barkın, Aran OT, Bahadır Ağce Z, Kayıhan H (2020). The Effects of Virtual Reality on Motor Functions and Daily Life Activities in Unilateral Spastic Cerebral Palsy: A Single-Blind Randomized Controlled Trial. Games Health J.

[57] Sandlund M, Domellöf E, Grip H, Rönnqvist L, Häger CK (2014). Training of goal directed arm movements with motion interactive video games in children with cerebral palsy - a kinematic evaluation. Dev Neurorehabil.

[58] Turconi AC, Biffi E, Maghini C, Peri E, Servodio Iammarone F, Gagliardi C (2016). Can new technologies improve upper limb performance in grown-up diplegic children?. Eur J Phys Rehabil Med.

[59] Weightman Andrew, Preston Nick, Levesley Martin, Holt Raymond, Mon-Williams Mark, Clarke Mike, Cozens Alastair J, Bhakta Bipin (2011). Home based computer-assisted upper limb exercise for young children with cerebral palsy: a feasibility study investigating impact on motor control and functional outcome. J Rehabil Med.

[60] Winkels DGM, Kottink AIR, Temmink RAJ, Nijlant JMM, Buurke JH (2013). Wii™-habilitation of upper extremity function in children with cerebral palsy. An explorative study. Dev Neurorehabil.

[61] Do J, Yoo E, Jung M, Park HY (2016). The effects of virtual reality-based bilateral arm training on hemiplegic children's upper limb motor skills. NeuroRehabilitation.

[62] Sandlund M, Waterworth EL, Häger Ch (2011). Using motion interactive games to promote physical activity and enhance motor performance in children with cerebral palsy. Dev Neurorehabil.

[63] Chang Y, Chen S, Huang J (2011). A Kinect-based system for physical rehabilitation: a pilot study for young adults with motor disabilities. Res Dev Disabil.

[64] Reifenberg G, Gabrosek G, Tanner K, Harpster K, Proffitt R, Persch A (2017). Feasibility of Pediatric Game-Based Neurorehabilitation Using Telehealth Technologies: A Case Report. Am J Occup Ther.

[65] Rios DC, Gilbertson T, McCoy SW, Price R, Gutman K, Miller KEF, Fechko A, Moritz CT (2013). NeuroGame Therapy to improve wrist control in children with cerebral palsy: a case series. Dev Neurorehabil.

[66] Robert M, Guberek R, Levin M, Sveistrup H (2013). Motor learning of the upper limb in children with cerebral palsy after virtual and physical training intervention.

[67] van Hedel H, Wick K, Meyer-Heim A, Eng K (2011). Improving dexterity in children with cerebral palsy.

[68] Dinomais M, Veaux F, Yamaguchi T, Richard P, Richard I, Nguyen S (2013). A new virtual reality tool for unilateral cerebral palsy rehabilitation: two single-case studies. Dev Neurorehabil.

[69] Golomb MR, Warden SJ, Fess E, Rabin B, Yonkman J, Shirley B, Burdea GC (2011). Maintained hand function and forearm bone health 14 months after an in-home virtual-reality videogame hand telerehabilitation intervention in an adolescent with hemiplegic cerebral palsy. J Child Neurol.

[70] Huber M, Rabin B, Docan C (2008). PlayStation 3-based tele-rehabilitation for children with hemiplegia.

[71] Qiu Q, Ramirez DA, Saleh S, Fluet GG, Parikh HD, Kelly D, Adamovich SV (2009). The New Jersey Institute of Technology Robot-Assisted Virtual Rehabilitation (NJIT-RAVR) system for children with cerebral palsy: a feasibility study. J Neuroeng Rehabil.

[72] Chang Y, Chen S, Huang J (2011). A Kinect-based system for physical rehabilitation: a pilot study for young adults with motor disabilities. Res Dev Disabil.

[73] Golomb MR, McDonald BC, Warden SJ, Yonkman J, Saykin AJ, Shirley B, Huber M, Rabin B, Abdelbaky Moustafa, Nwosu ME, Barkat-Masih M, Burdea GC (2010). In-home virtual reality videogame telerehabilitation in adolescents with hemiplegic cerebral palsy. Arch Phys Med Rehabil.

[74] Charles J, Gordon AM (2006). Development of hand-arm bimanual intensive training (HABIT) for improving bimanual coordination in children with hemiplegic cerebral palsy. Dev Med Child Neurol.

[75] Gordon AM, Charles J, Wolf SL (2005). Methods of constraint-induced movement therapy for children with hemiplegic cerebral palsy: development of a child-friendly intervention for improving upper-extremity function. Arch Phys Med Rehabil.

[76] Nudo R (2003). Adaptive plasticity in motor cortex: implications for rehabilitation after brain injury. J Rehabil Med.

[77] Demers M, Mbiya N, Levin M (2017). Industry and academia collaboration in the design of virtual reality applications for rehabilitation.

[78] Winstein CJ, Schmidt RA (1990). Reduced frequency of knowledge of results enhances motor skill learning. J Exp Psychol Learn Mem Cogn.

[79] Laufer Y, Weiss P L (2011). Virtual reality in the assessment and treatment of children with motor impairment: A systematic review. J Phys Ther Educ.

[80] Sigrist R, Rauter G, Riener R, Wolf P (2013). Augmented visual, auditory, haptic, and multimodal feedback in motor learning: a review. Psychon Bull Rev.

[81] Chiviacowsky S, Wulf G, de Medeiros FL, Kaefer A, Wally R (2008). Self-controlled feedback in 10-year-old children: higher feedback frequencies enhance learning. Res Q Exerc Sport.

[82] Hemayattalab R, Arabameri E, Pourazar M, Ardakani MD, Kashefi M (2013). Effects of self-controlled feedback on learning of a throwing task in children with spastic hemiplegic cerebral palsy. Res Dev Disabil.

[83] Subramanian SK, Massie CL, Malcolm MP, Levin MF (2010). Does provision of extrinsic feedback result in improved motor learning in the upper limb poststroke? A systematic review of the evidence. Neurorehabil Neural Repair.

[84] Schüler T, Ferreira dos Santos L, Hoermann S (2015). Designing virtual environments for motor rehabilitation: Towards a framework for the integration of best-practice information.

[85] Guadagnoli MA, Lee TD (2004). Challenge point: a framework for conceptualizing the effects of various practice conditions in motor learning. J Mot Behav.

[86] Metcalfe J (2017). Learning from Errors. Annu Rev Psychol.

[87] Levac DE, Miller PA (2013). Integrating virtual reality video games into practice: clinicians' experiences. Physiother Theory Pract.

[88] Majnemer A (2011). Importance of motivation to children's participation: a motivation to change. Phys Occup Ther Pediatr.

[89] Meyns P, Roman de Mettelinge T, van der Spank J, Coussens M, Van Waelvelde H (2018). Motivation in pediatric motor rehabilitation: A systematic search of the literature using the self-determination theory as a conceptual framework. Dev Neurorehabil.

[90] Majnemer A, Shikako-Thomas K, Lach L, Shevell M, Law M, Schmitz N, QUALA group (2013). Mastery motivation in adolescents with cerebral palsy. Res Dev Disabil.

[91] Tatla SK, Sauve K, Jarus T, Virji-Babul N, Holsti L (2014). The effects of motivating interventions on rehabilitation outcomes in children and youth with acquired brain injuries: a systematic review. Brain Inj.

[92] Harris K, Reid D (2005). The influence of virtual reality play on children's motivation. Can J Occup Ther.

[93] Kitago T, Krakauer J, Barnes MP, Good DC (2013). Motor learning principles for neurorehabilitation. Handbook of Clinical Neurology. Vol 110.

[94] Levac DE, Huber ME, Sternad D (2019). Learning and transfer of complex motor skills in virtual reality: a perspective review. J Neuroeng Rehabil.

[95] Yanovich E, Ronen O (2015). The use of virtual reality in motor learning: A multiple pilot study review. APE.

[96] Breslin G, Hodges NJ, Steenson A, Williams AM (2012). Constant or variable practice: recreating the especial skill effect. Acta Psychol (Amst).

[97] Lage GM, Ugrinowitsch H, Apolinário-Souza Tércio, Vieira MM, Albuquerque MR, Benda RN (2015). Repetition and variation in motor practice: A review of neural correlates. Neurosci Biobehav Rev.

[98] Bernstein N (1968). The Co-ordination and Regulation of Movements.

[99] Guadagnoli MA, Holcomb WR, Weber TJ (1999). The relationship between contextual interference effects and performer expertise on the learning of a putting task. J Hum Mov Stud.

[100] Zipp GP, Gentile AM (2010). Practice Schedule and the Learning of Motor Skills in Children and Adults: Teaching Implications. Journal of College Teaching & Learning.

[101] Pinto-Zipp G, Gentile A (1995). Practice schedule in motor learning: children versus adults.

[102] Levin M, Sveistrup H, Subramanian S (2010). Feedback and virtual environments for motor learning and rehabilitation. Schedae.

