# Supplementary material for: Integration of Motor Learning Principles Into Virtual Reality Interventions for Individuals With Cerebral Palsy: Systematic Review
Source: JMIR Serious Games. 2021 Apr 7;9(2):e23822. doi: 10.2196/23822 (PMC8060861; doi:10.2196/23822)
Supplement: Multimedia Appendix 1 [file games_v9i2e23822_app1.pdf]

Supplementary material

Detailed search strategy

MEDLINE search

Number	Key terms
1	Virtual Reality/ OR Therapy, Computer-Assisted/ OR Video Games/ or (virtual reality OR vr OR video game* OR serious game* OR active video game* OR gaming OR exergaming).ti,ab,kf,kw,jw
2	Rehabilitation/ OR Physical and Rehabilitation Medicine/ OR Occupational Therapy/ OR Exercise Therapy/ OR Physical Therapy Modalities/ OR Physical Therapy Specialty/ OR Therapeutics/ OR (physiotherapy OR physical therapy OR treatment OR intervention).ti,ab,kf,kw,jw
3	Cerebral palsy/ OR (cerebral pals* OR brain pals* OR brain paralysis OR central paralysis OR cerebral paralysis OR cerebral paresis OR encephalopathia infantilis OR little disease OR little's disease OR spastic diplegia*).ti,ab,kf,kw,jw
4	1 AND 2 AND 3 AND (english or french).lg

EMBASE search

Number	Key terms
1	video game/ OR virtual reality/ OR (virtual reality OR vr OR video game* OR serious game* OR active video game* OR gaming OR exergaming).ti,ab,kw,jx
2	rehabilitation/ OR occupational therapy/ OR physiotherapy/ OR (Treatment OR intervention).ti,ab,kw,jx
3	Cerebral palsy/ OR (cerebral pals* OR brain pals* OR brain paralysis OR central paralysis OR cerebral paralysis OR cerebral paresis OR encephalopathia infantilis OR little disease OR little's disease OR spastic diplegia*).ti,ab,kw,jx
4	1 AND 2 AND 3
5	limit 4 to (english or french)
6	5 AND Upper Extremity/ OR Arm/ OR (Upper Extremit* or Arm* or Hand* or Upper limb*).ti,ab,kw,jw.

CINAHL

Number	Key terms
1	(MH "Virtual Reality Exposure Therapy") OR (MH "Virtual Reality") OR TI(gaming OR exergam* OR serious OR game* video game* OR vr) OR AB(gaming OR exergam* OR serious OR game* video game* OR vr)
2	(MH "Physical Therapy") OR (MH "Occupational Therapy Practice, Research-Based") OR (MH "Research, Physical Therapy") OR (MH "Research, Occupational Therapy") OR "rehabilitation or therapy or treatment" OR (MH "Pediatric Physical Therapy") OR (MH "Pediatric Occupational Therapy")
3	MH("Cerebral palsy") OR TI(cerebral pals* OR brain pals* OR brain paralysis OR central paralysis OR cerebral paralysis OR cerebral paresis OR encephalopathia infantilis OR little disease OR little's disease OR spastic diplegia*) OR AB(cerebral pals* OR brain pals* OR brain paralysis OR central paralysis OR cerebral paralysis OR cerebral paresis OR encephalopathia infantilis OR little disease OR little's disease OR spastic diplegia*)
4	1 and 2 and 3 AND LA (english OR french)

Web of science

Number	Key terms
1	((virtual reality or video game* or serious games or active video game* or gaming or exergam*) and (rehabilitation or occupational therapy or physical therapy or physiotherapy) and cerebral palsy)<br>All field selected

OT seeker

Number	Key terms
1	(virtual reality) and (cerebral palsy)
2	(Video games) and (cerebral palsy)
3	(exergaming) and (cerebral palsy)
	1 or 2

PEDro

Number	Key terms
1	virtual reality and cerebral palsy
2	Video game and cerebral palsy
3	exergaming and cerebral palsy
	1 or 2 (excluding systematic reviews)

SCOPUS

Number	Key terms
1	((*"virtual reality"* OR *"video game*"* OR *"exergam*")* AND (OR *"occupational therapy"* OR *"physical therapy"* OR *"physiotherapy")* AND *"cerebral palsy"*) AND (LIMIT-TO (LANGUAGE , *"English"*) OR LIMIT-TO (LANGUAGE , *"French"*))

Cochrane Database, the Cochrane Central Register of Controlled Trials

Number	Key terms
1	Virtual reality therapy
2	Virtual reality

IEEE

Number	Key terms
1	Virtual reality and cerebral palsy

Supplementary Table 1 - Incorporation of motor learning principles in the 26 studies retrieved

Ref	Frequency Intensity Duration	Movement repetition (per session)	Task-specific practice	Type of practice	Difficulty Progression	Feedback Modality<br><br>Feedback Delivery Schedule	Assessment of Motivation	Outcome Measures of UL function	Outcome of VR Intervention	Assessment of Retention	Assessment of Transfer of Skills<br><br>Skills Transferred
[44]	3 sessions of 60 min/week for 8 weeks, 1440 min in total	Not specified	Simulated sports, reach and grasp movement, functionally relevant	Variable	Yes. For the custom games, the therapist selected the game level based on speed and success rate.	Haptic, Auditory and Visual<br><br>Continuous	No	MMDT, Grip strength, Duruoz Hand Index	MMDT: Significant improvement post-intervention (effect size = 0.64)<br>Grip strength: Significant improvement on the less- and more-affected UL<br>Duruoz Hand Index: Significant improvement in fine motor ability	No	Yes, with the Childhood Health Assessment Questionnaire<br><br>Dressing, eating, hygiene, grip, reach
[45]	30 min, 3 days/week for 4 months, 1080 min in total	Not specified	Simulated sports, functionally relevant	Variable	No	Auditory, Visual<br><br>Continuous	No	ABILHAND-Kids, PDMS	Object manipulation, visual-motor skills and upper limb functions significantly improved in the intervention group.	No	No
[61]	1 session / day, Phase 1: 8 days,	45	Limited, repetitive movement	Variable	No	Auditory, Visual	No	Ability to perform the	Significant improvement in the number of	No	No

	Phase 2: 7 days, 15 days in total, time not specified		(hand up, hand forward and hand to mouth), not functionally relevant			Continuous		correct movement	correct UL movements.		
[72]	2-3 sessions/ week for 4 weeks, 480 min in total	100 repetitions for 3 minutes of play of VR 2	Reach and grasp movement, functionally relevant	Variable and massed practice	Yes, VR 1: Adjustable difficulty levels included in the VR system (speed, object size, target location, object moving path); VR 2: decided by the clinician based on task success (progression based on 3 speeds)	Auditory, Visual, and Haptic for VR 2 only<br><br>Continuous	Yes, observation: Increased motivation with intervention, Varied from one participant to another (from poor to good)	PDMS-2 (Fine Motor Domain) Reaching kinematics (movement time, path length, peak velocity, and movement units),	PDMS-2: All 4 participants showed improvement ranging from 1-11 points. *Neutral direction:* Post-intervention, Participant 4 had faster and straighter reaches, Participant 2 had more forceful reaches, and Participants 1 and 4 had smoother reaches. *Outward direction:* Post-intervention, Participants 1 and 2 had straighter reaches, Participant 4 had more forceful reaches, Participant 1 showed smoother reaches. *Inward*	Yes, 2 participants showed an increase on the PDMS-2 at follow-up. 1 participant showed an unchanged score.	No

									direction: Participant 1 showed faster reaches, Participants 1 and 4 had smoother reaches.		
[48]	3 days/week for 3 weeks, time not specified	Not specified	Simulated sports, functionally relevant	Variable	No	Auditory, Visual<br><br>Continuous	Yes, observation: Increased motivation with intervention	Manual Ability Classification System	The effect of VR was not significant on manual ability score compared to the control group.	No	No
[60]	2 sessions/ day, 5 days/week for 2 weeks, 1200 min in total	Not specified	Functional catching movement	Not specified	Yes, the properties of the virtual object (e.g., direction, velocity, size, shape), the movement amplitude and feedback provision (control/ display ratio) can be changed	Visual<br><br>Continuous	No	BBT, Jebsen	BBT: Improvement of the gross manual dexterity of the less and the more affected UL;<br>Jebsen: Improvement on the ''full cans moving'' sub-test of the Jebsen for 1 participant.	No	No
[49]	30 minutes over 12 sessions, 360 min in total	Not specified	Simulated sports, functionally relevant	Variable	No	Auditory, Visual<br><br>Continuous	No	WMFT, Pediatric MAL, Coordination testing (affected	WMFT: All participants showed improvements on the performance	Yes, retention was observed	No

								side and bilateral via putting a basketball through the hoop, moving large lightweight boxes)	scores and times. MAL: Amount of use and movement quality of the affected upper limb improved post-intervention. Coordination testing: the number of successful basketball shots increased and the time taken to move boxes decreased in all subjects		
[55]	45 min, 3 days/weeks for 12 weeks, 1620 min in total	Not specified	Task-oriented movement, functionally relevant	Variable	Yes, ranges of motion, resistance or gravity-assistance, accuracy, speed, levels of difficulty determined by the system or the therapist (very easy, easy, moderate, difficult)	Auditory, Visual<br><br>Continuous	No	QUEST, Modified Ashworth Scale	Armeo robotic therapy leads to greater improvement in the modified Ashworth Scale and QUEST scores of the affected UL than the conventional therapy	No	No
[67-	60 min, 3	40	Game-like	Variable	Yes,	Haptic,	No, but	Grip and	All participants	No	No

71]	days/week for 3 weeks, 540 min in total (24 min of active time /session)		simulations, functionally relevant	and massed practice	ranges of motion, resistance or gravity assistance , speed and accuracy, number of objects, height, width and distance of the target	Auditory, Visual<br><br>Continuous	subjective feedback by participants noted	pinch strength, Melbourn e, Ranges of motion, Functional Level of Hemiplegi a scale, Reaching kinematic s	improved in movement speed, trajectory smoothness and efficiency. Melbourne: the group of 9 participants improved post-intervention, Shoulder flexion and grip strength improved for the group in which VR training was combined with constraint induced movement therapy.		
[66]	2.6 sessions a week for an average of 21 min/ session, 14 months, 2931 min in total	Not specified	Game-like simulation, repetitive movement of fingers, not functionally relevant	Variable	Yes, ranges of motion and movemen t/target speed; customize d threshold s, 3 levels: easy, medium, hard	Auditory, Visual<br><br>Continuous	Yes, Increased motivation observed with interventio n (subjective assessment)	Jebsen, Grip Strength, Forearm bone health	Improvements in grip strength and hand function post-intervention	No	No
[64]	60 minutes/we ek (mean of 23 minutes/ session for wrist extension	550	Limited, game-like simulation, repetitive movements	Variable and massed practice	Yes, weight resistance and range of movemen t	Haptic, Visual (game screen)<br><br>Continuous	Yes, semi-structured interview: Increased motivation with interventio	Active range of motion of the wrist	Range of motions: Gains in wrist extension speed, but not wrist range of motion	No	Yes, measured with the COPM<br><br>All children made

	games, 17 minutes/ session for elbow/ shoulder games), 12 weeks, 720 min in total				thresholds adjusted by the therapist based on child's energy level (the range-of-motion setting was seldom adjusted)		n, Motivation highly variable between participants				significant progress toward their established goals.
[65]	30 min/, 5 days/week, 3 months, 774 to 1506 min in total	Not specified	Game-like simulation, repetitive movement of fingers, not functionally relevant	Variable and random practice	Yes, ranges of motion and movement/target speed; customized thresholds, 3 levels: easy, medium, hard	Auditory, Visual, KP and KR<br><br>Continuous	Yes, spontaneous feedback: Increased motivation with intervention, Games designed to maintain child's interest (random images, different options of target to grasp)	BOT, Jebsen, grip and pince strength, ranges of motion from the Ultra Glove, forearm bone health	Improvement on grip strength and the Jebsen, no changes for the BOT, changes in ROM for some fingers for some participants	No	No
[50]	30min, 2 days/week for 6 weeks, 360 min. in total, moderate intensity reported	Not specified	Game-like simulation involving object manipulation (not further specified)	Variable	No	Auditory, Visual<br><br>Continuous	Yes, user satisfaction questionnaire: Increased motivation with intervention	Melbourne	2 participants in the intervention group improved by 9% and 13%. Other children did not change	No	No
[51]	40 min, 5 days/week for 6 weeks,	~144	Simulated sports, functionally	Variable	No	Haptic, Auditory, Visual	Yes, logbooks and	ABILHAND-kids, grip	Improvements were minimal: 2 participants	Yes, retention was	No

	1080 to 1440 min in total		relevant			Continuous	questionnaires: Increased motivation with intervention, higher compliance and more consistently positive responses to motivation and feasibility questions than strength training group	strength, Melbourne	improved on the Melbourne, 1 participant improved on grip strength post-intervention	observed at 4-week follow-up	
[63]	60 min/week for 5 weeks, 300 min in total	Not specified	Limited, not functionally relevant	Variable	Unclear	Visual<br>Continuous	No	Therapists feedback on perceived usefulness and observed improvements, functional tasks with Velcro strips and a deck of cards	Therapists perceived that participants' reaching ability improved post-intervention, participants improved in their ability to complete the functional tasks.	No	No
[68]	60 min, 3 days/week for 3 weeks, 540 min in total	Not specified	Game-like simulations, functionally relevant	Variable	Yes, Ranges of motion, resistance training or gravity assistance, speed	Haptic, Auditory, Visual<br>Continuous	Yes, questionnaire: varied from one game to another	Active ROM, Melbourne, Reaching kinematics (movemen	Strength: Both participants had an almost 100% increase on strength tests. Kinematics: Both	No	No

					and accuracy, number of objects, height, width and distance of the target			t speed and smoothness) Strength	participants showed improvement on several kinematic measures Melbourne: One participant showed improvements in overall performance. The second subject made improvements in upper extremity active range of motion and in kinematic measures of reaching movements.		
[58]	480 min/week for 7 weeks, 3360 min in total	Not specified	Game-like simulations, functional movements	Constant	Unclear	Visual<br><br>Continuous	Yes, interview: Increased motivation with intervention	AHA, BOT, Pediatric MAL, PEDI-CAT, QUEST	Performance improved for all outcome measures from pre- to posttest with the exception of the QUEST Dissociated Movements subscale, which remained the same.	No	No
[69]	Protocol 1: Lab: 10 sessions Home: 30 min, 3 days/week for 3-4 weeks	Not specified	Limited, not functionally relevant	Constant	Yes, Modification of gain values to facilitate selective activation of specific	Visual<br><br>Continuous	No	sEMG during game play and during active movement, active	Participants demonstrated dramatic improvement of the sEMG activity during game play. Several	Yes, retention did not occur	No

	Protocol 2: Lab: 3 sessions over 1 week Home: 30 min, 3 days/ week for 4 weeks, 528 min in total				muscles			ROM, Shriner's Hospital Upper Extremity Evaluation	participants also showed improvements in range of motion, co-contraction, and spontaneous upper extremity function following the VR intervention.		
[57]	60 min, 3 days/week for 5 weeks, 900 min in total (20 min of VR activities/ session)	Not specified	Functionally relevant reach-to-grasp task	Not specified	Unclear	Visual<br><br>Continuous	No	Melbourne, Sensory assessment, ROM, reaching kinematic (shoulder and elbow range of motion, endpoint straightness, and endpoint velocity)	Improvements of UL kinematics led to better learning and retention of movement patterns	Yes, motor improvements were retained 3 months post-intervention for all 4 kinematic variables	Yes, measured in a similar task performed in the physical environment<br><br>Motor improvements were transferred to a similar task in ~2/3 of the children for all kinematic variables.
[56]	90 min, 3 days/week for 4 weeks, 1080 min in total	Not specified	Limited, game-like simulation, repetitive movement, not functionally relevant	Variable	Yes, Tool, load, range of motion, time duration, and speed and difficulty	Auditory, Visual<br><br>Continuous	No	BOT, Pediatric MAL	Children who received CIMT in VE had greater improvement in the amount of use, quality of movements, and speed and	Yes - retention was observed at 3 month follow-up	No

					of exercises				dexterity of the affected UL vs. children who received the VR, modified CIMT, or conventional therapy		
[62]	45 min/session, 2 days/week for 8 weeks, 720 min in total	Not specified	Game-like simulations, functionally relevant	Variable	Unclear, difficulty levels varied according to each child's performance	Visual<br><br>Continuous	No	BOT	BOT fine motor function significantly improved post-intervention (effect size: 1.56). Improvements were greater for the VR group than the traditional occupational therapy group	No	No
[52-54]	A minimum of 20 min/day (range from 22-52) for 4 weeks, 560 min in total	Not specified	Functionally relevant	Variable	Unclear	Auditory, Visual, KR: total score<br><br>Continuous	Yes, semi-structured interviews: Increased motivation with intervention	BOT, Movement ABC-2, Kinematics (movement smoothness and precision)	Post-intervention, decreased peak velocity, straighter movements, increased movement precision, less variation in shoulder angles and a reduced center of pressure path in VE	Not assessed	Yes, measured in a similar task performed in the physical environment<br><br>Improvement in movement smoothness in a similar reaching task in the physical environment post-intervention
[46]	45 min, 2 sessions/da	Not specified	Functionally relevant	Variable	Yes, based on	Auditory, Visual, KP	Yes, Increased	Ashworth Scale,	UL coordination,	No	Yes, measured

	y, 40 sessions in total over 4 weeks, 1800 min in total				an algorithm	and KR, therapist verbally reinforced positive efforts<br><br>Continuous	motivation with intervention	Melbourne, QUEST, ROM, finger tapping	fluency and quality of movements mainly of hands and fingers significantly improved. No significant change of ROM, in spasticity or QUEST score.		with an experimental parent/patient questionnaire<br><br>Good transferability to everyday life also in areas not specifically trained, such as self-care abilities and mobility.
[59]	45 min, 3 days/week for 4 weeks, 1620 min in total	Not specified	Game-like simulations, functionally relevant	Variable	Yes, Adjusted by the therapist	Haptic, Visual<br><br>Continuous	No	BBT, Nine Hole Peg Test, Grip Strength, Pince Strength	The BBT tended to improve more in the intervention compared to the control group (P = 0.07). Effect sizes of most measures were considerably larger in the intervention group.	No	No
[70]	Unrestricted use for 4 weeks, mean of 75 min in total (range: 0.2–271 min)	Mean of 606 outward and 734 inward movement in total	Limited, game-like simulations, aiming movement	Variable and massed	Yes, range of motion, grasping ability, physical assistance given by the joystick determin	Haptic, Visual<br><br>Continuous	No	Kinematic analysis	Movement time, peak velocity and movement jerkiness improved post-intervention	No	Yes, COPM<br><br>Improvement in some of these personal activities of daily living post-intervention

					ed by an algorithm						
[47]	30 min, 2 days/week for 6 weeks, 360 min in total	Not specified	Simulated sports, functionally relevant	Variable	No	Auditory, Visual<br><br>Continuous	Yes, visual analogue scale: motivation with the intervention, varied between games and children	ABILHAND-Kids, Melbourne	The quality of upper extremity movements did not change (no significant change on the Melbourne), while a significant increase of in using hands/arms during 2 daily activities was found on the ABILHAND.	No	No

[a]Abbreviations: AHA: Assisting Hand Assessment, BBT: Box and Blocks tests, BOT: Bruininks-Oseretsky Test of Motor Proficiency, CIMT: Constraint-induced movement therapy, COPM: Canadian Occupational Performance Measure, Jebsen: Jebsen Hand Function Test, KP: Knowledge of performance, KR: knowledge of results, MAL: Motor Activity Log, Melbourne: Melbourne Assessment of Unilateral Upper Limb Function, min: minutes, MMDT: Minnesota Manual Dexterity Test, n: sample size, PDMS: Peabody Developmental Motor Scales, PEDI-CAT: Pediatric Evaluation and Disability Inventory—Computer Adapted Test, QUEST: Quality of Upper Extremity Skills Test, RCT: Randomized control trial, sEMG: Surface Electromyography, UL: Upper limb, VE: virtual environment, VR: virtual reality, WMFT: Wolf Motor Function Test
